# The spatial structure of resting state connectivity stability on the scale of minutes

**DOI:** 10.3389/fnins.2014.00138

**Published:** 2014-06-11

**Authors:** Javier Gonzalez-Castillo, Daniel A. Handwerker, Meghan E. Robinson, Colin Weir Hoy, Laura C. Buchanan, Ziad S. Saad, Peter A. Bandettini

**Affiliations:** ^1^Section on Functional Imaging Methods, Laboratory of Brain and Cognition, National Institute of Mental Health, National Institutes of HealthBethesda, MD, USA; ^2^Translational Research Center for TBI and Stress Disorders (TRACTS), VA Boston Healthcare SystemBoston, MA, USA; ^3^Scientific and Statistical Computing Core, National Institute of Mental Health, National Institutes of HealthBethesda, MD, USA

**Keywords:** fMRI, connectivity dynamics, stability, rest, sliding window analysis

## Abstract

Resting state functional MRI (rsfMRI) connectivity patterns are not temporally stable, but fluctuate in time at scales shorter than most common rest scan durations (5–10 min). Consequently, connectivity patterns for two different portions of the same scan can differ drastically. To better characterize this temporal variability and understand how it is spatially distributed across the brain, we scanned subjects continuously for 60 min, at a temporal resolution of 1 s, while they rested inside the scanner. We then computed connectivity matrices between functionally-defined regions of interest for non-overlapping 1 min windows, and classified connections according to their strength, polarity, and variability. We found that the most stable connections correspond primarily to inter-hemispheric connections between left/right homologous ROIs. However, only 32% of all within-network connections were classified as most stable. This shows that resting state networks have some long-term stability, but confirms the flexible configuration of these networks, particularly those related to higher order cognitive functions. The most variable connections correspond primarily to inter-hemispheric, across-network connections between non-homologous regions in occipital and frontal cortex. Finally we found a series of connections with negative average correlation, but further analyses revealed that such average negative correlations may be related to the removal of CSF signals during pre-processing. Using the same dataset, we also evaluated how similarity of within-subject whole-brain connectivity matrices changes as a function of window duration (used here as a proxy for scan duration). Our results suggest scanning for a minimum of 10 min to optimize within-subject reproducibility of connectivity patterns across the entire brain, rather than a few predefined networks.

## Introduction

In recent years, the functional magnetic resonance imaging (fMRI) research community has undertaken a slow, yet constant shift in attention from functional localization (where in the brain a specific function resides) to functional connectivity (how different brain regions interact with each other). Today, it is well established that some brain regions are tuned primarily to perform specific tasks (e.g., motor cortex controls the movement of body parts, visual cortex analyzes incoming visual stimuli, etc.) Still, this one-to-one relationship soon diffuses as one moves beyond primary cortices into association cortex to understand the neuronal correlates of higher cognitive functions such as emotions, speech, or attention. Moreover, it is increasingly common to discover variations in functional connectivity, rather than in specific functional modules, that seem to differentiate complex mental conditions (see Greicius, 2008 for a review) such as autism (Just et al., 2007; Gotts et al., 2012), depression (Sheline et al., 2010), and Alzheimer's Disease (Wang et al., 2013a).

One well-known, non-invasive approach to the study of functional connectivity in the human brain is resting state fMRI (rsfMRI; Biswal et al., 1995). In this technique, the spatial co-fluctuation of Blood Oxygenation Level Dependent (BOLD) signals is recorded while subjects rest quietly in the scanner in the absence of any specific task demands, and these data are used to explore patterns of functional connectivity at the system level (see Lowe, 2010 for a historical review). More importantly, rsfMRI is not only a powerful research tool, but it has great potential for clinical applications given its experimental simplicity, short scanning durations, richness of information, ease of sharing, and low requirement for subject compliance. Nevertheless, for clinicians to be able to rely on rsfMRI-based biomarkers to diagnose or intervene, several challenges with respect to reproducibility and interpretation must be resolved (Castellanos et al., 2013). Although overall patterns of rsfMRI-based functional connectivity have proven to be reliable across scans, subjects, and even institutions, quantitative measures with the potential to become biomarkers (e.g., the strength of a given connection) are not yet sufficiently reliable, as they depend on factors such as scan condition (e.g., eyes closed vs. eyes open Yan et al., 2009; Van Dijk et al., 2010; McAvoy et al., 2012), scan duration (Birn et al., 2013), and specific pre-processing steps used during the analysis (Murphy et al., 2009; Power et al., 2012). Despite these dependences, some rsfMRI connectivity metrics such as regional homogeneity (ReHo; Zuo et al., 2013), amplitude of spontaneous low frequency oscillations (Zuo et al., 2010), and several measures of centrality (Zuo et al., 2012) have been shown to have encouraging test–retest reliability. Nevertheless, one additional factor that poses interesting questions regarding how to best record and quantify rsfMRI-based metrics is the recently observed dynamic behavior of rsfMRI connectivity patterns (Chang and Glover, 2010).

Several recent studies have shown how patterns of rsfMRI connectivity vary substantially even over the duration of a single scan (Chang and Glover, 2010; Handwerker et al., 2012; Tagliazucchi et al., 2012; Hutchison et al., 2013b), thereby calling into question the assumption of temporal stationarity even over short timescales (see Hutchison et al., 2013a for a review). Similarly, other studies have explored how scan duration affects the reproducibility of rsfMRI connectivity patterns (Van Dijk et al., 2010; Birn et al., 2013). However, most of these studies have focused their analysis on a handful of representative connections and networks. Given the large variability of functional roles and connection strengths across the human brain connectome, it can be expected that optimal scan acquisition strategies and reliability of biomarker measurements will depend greatly on the connections of interest. For example, Allen et al. (2014) recently reported a series of rsfMRI networks, labeled the “Zone of Instability,” that exhibit significantly greater temporal variability in functional connectivity. These regions with the greatest instability correspond primarily to dorsal attention areas, default mode regions, and superior occipital areas. Still Allen and colleges' exploration of dynamic behavior was constrained by the duration of the resting scans (5 m and 4 s) and their temporal resolution (2 s), which limit both the quality of functional connectivity estimates (given the low number of available data points) and the domain of functional connectivity configurations that occur during such short scan periods.

The purpose of the current study is to further explore and characterize rsfMRI connectivity dynamics, and in that manner extend some of the findings of Allen et al. (2014) and others (Tagliazucchi et al., 2012; Hutchison et al., 2013b). To overcome the above-mentioned limitations resulting from short scan durations, in this study rsfMRI data were collected in 12 participants who were scanned continuously for 60 min at a temporal resolution of 1 s. Using these data, we evaluated pair-wise connections over the scale of minutes, investigating their polarity, strength, and variability. We evaluated the spatial distribution of three categories of connections (namely stable positive connections, variable positive connections, and negative connections) and whether assignment of connections to these three groups was consistent across subjects. Using a sliding window approach, we found that most stable positive connections correspond mainly to symmetric, inter-hemispheric, within- and across-network connections; while most variable positive connections correspond primarily to inter- and intra-hemispheric, across-network connections between occipital and frontal regions. Negative connections correspond primarily to those between two medial subcortical regions and fronto-parietal regions. We also evaluated how window length, a proxy for scan duration, affects the degree of similarity in whole-brain, within-subject connectivity patterns. We found two regimes in terms of how similarity changes with scan duration. For short scan durations (approximately less than 10 min) similarity of whole-brain connectivity patterns decreases quickly as scan duration shortens. For longer durations, although similarity increases with scan length, it does so at a much lower rate.

## Materials and methods

### Data acquisition

Twelve healthy volunteers (7 males; age: 30.17 ± 10.22 years) participated in this study after providing written consent in agreement with a protocol approved by the NIH Protocol Review Board. Subjects were scanned continuously in a General Electric 3T MRI scanner for 60 min while relaxing with their eyes closed. A 32-channel receive-only head coil was used. The resting scans were acquired using a gradient-recalled echo-planar imaging (EPI) sequence (*TR* = 1 s, *TE* = 27 ms, FOV = 24/21 cm, image matrix = 64 × 64/72 × 72, slice thickness = 4.0 mm, slice spacing = 0.0 mm, flip angle = 60°, number of slices = 23, number of acquisitions = 3600, ASSET Acceleration = 2). In addition, a high-resolution T1 spoiled gradient echo (SPGR) scan was acquired for alignment and presentation purposes (sagittal prescription, number of slices per slab = 176, slice thickness = 1 mm, FOV = 256 mm, image matrix = 256 × 256) in each subject.

Respiration and cardiac traces were also collected during the resting scans using a respiration belt and a pulse oximeter, in all subjects except one. Both physiological traces were acquired with a sampling rate of 50 Hz.

In order to achieve a temporal resolution of 1 s, it was necessary to restrict our spatial coverage. In particular, with the current data, we cannot draw any conclusions regarding connections involving the cerebellum, temporal poles, or ventral temporal regions. New technological developments, such as multi-slice acquisition techniques (Feinberg and Setsompop, 2013), may soon be able to eliminate this limitation (Smith et al., 2012).

### Data pre-processing

Data pre-processing was conducted with the AFNI software package (Cox, 1996). Pre-processing steps include: discarding of initial 10 volumes to allow for magnetic homogenization; despiking (with AFNI *3dDespike*); physiological noise correction (in all subjects but one) including regressors for the RETROICOR (Glover et al., 2000), RVT (Birn et al., 2006), and RHR (Chang et al., 2009) models; slice time correction (AFNI program *3dTshift*); head motion correction (AFNI program *3dvolreg*) and transformation into MNI space (AFNI program *@auto_tlrc*) in a single interpolation step; and spatial smoothing (FWHM = 6 mm). In addition, mean, linear trends, signal from local white matter (WM), signal from the lateral ventricles (CSF), motion estimates, the first derivative of motion estimates, and a series of sine and cosine functions to remove all frequencies outside the range (0.01–0.25 Hz) were regressed out in a single regression step (AFNI program *3dTproject*). This last regression step permits us to account for potential hardware instabilities and remaining physiological noise (ANATICOR; Jo et al., 2010, 2013; Gotts et al., 2013). During this regression step, time points with motion greater than 0.4 mm were removed from the data (scrubbing) and replaced by values obtained via linear interpolation in time. On average, 1649 degrees of freedom (DOF) remain after the above-mentioned regression and censoring steps (Table 1 shows motion, number of interpolated volumes, and remaining DOFs for each subject).

**Table 1:** **Motion, number of censored time points, and remaining DOFs after bandpass filtering, regression of nuisance signals, and censoring in each subject**.

	**Max. absolute displacement (mm)**	**Max. relative displacement (mm)**	**# Data points interpolated**	**Remaining DOF**
SBJ01	5.07	0.92	13	1694
SBJ02	5.16	1.10	14	1693
SBJ03	4.12	0.65	40	1667
SBJ04	5.73	0.50	2	1705
SBJ05	1.97	0.31	0	1707
SBJ06	1.99	0.30	0	1707
SBJ07	4.52	0.68	2	1705
SBJ08	2.59	0.24	0	1707
SBJ09	2.91	0.47	452	1255
SBJ10	6.62	0.80	82	1625
SBJ11	3.60	0.26	0	1707
SBJ12	3.71	1.05	88	1619
Mean	4.00	0.61	57.75	1,649.25

*Participant SBJ09 was excluded from all sliding window analyses due to the large number of data points that required interpolation due to head movement according to the criteria set during pre-processing*.

Spatial transformation matrices to go back and forth between the original EPI space, T1-anatomical space, and MNI standard space were also computed for each subject using AFNI programs *3dAllineate* and *@auto_tlrc*. These matrices were subsequently used for presentation purposes and to bring publicly available atlases into each subject's functional data space (see below).

### Brain parcellation

**Figure 1:**
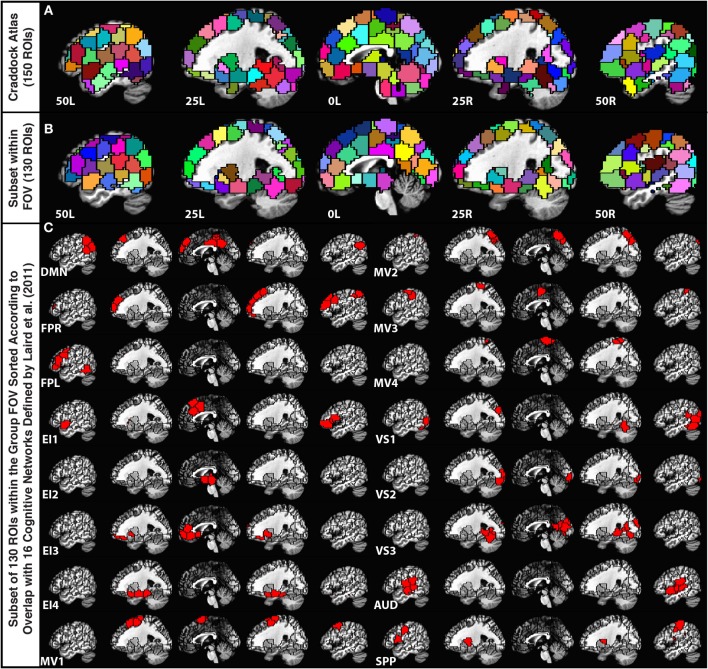
**(A)** Depiction of the 150-ROI Craddock Atlas on top of five sagittal slices in the MNI stereotaxic space. **(B)** Depiction of the remaining 130 ROIs from the atlas considered in this study. ROIs eliminated from the original atlas correspond mainly to the cerebellum and inferior temporal regions that were not part of the imaging FOV for all 12 participants. **(C)** Grouping of the remaining ROIs according to the Laird et al. (2011) functional network templates.

In order to parcellate the brain into a limited number of spatially contiguous, functionally homogeneous, non-overlapping regions of interest (ROIs), we used the publicly available template of 150 ROIs associated with the Craddock Atlas (Craddock et al., 2012) (Figure 1A). An ROI-based approach was selected over a voxel-wise approach to help with interpretation, minimize the contribution of small errors in alignment to between-subject comparisons, and ease computational load. Nevertheless, despite using a functionally-based atlas with relatively small ROIs, some level of functional inhomogeneity should be expected when combining voxels into a single time-series (Zuo et al., 2013).

For each subject, we first brought this MNI atlas template into each subject's EPI space. Subsequently, we removed ROIs (20 ROIs from cerebellum, midbrain, and lower temporal cortex) that did not have at least 10 voxels within the imaged field of view for all 12 subjects (Figure 1B).

In order to group the remaining 130 non-overlapping ROIs into functionally relevant networks, we used the functional network taxonomy published by Laird et al. (2011), excluding two artifactual networks (ICNs 19 and 20 identified as artifactual by Laird and colleagues) and two networks not covered by our scanning FOV (ICNs 5 and 14). Each ROI was assigned to one of the 16 remaining networks described by Laird and colleagues by identifying the network with maximal spatial overlap with that ROI (Figure 1C). Within each network, ROIs in connectivity matrices appear sorted according to decreasing degree of overlap with that network. Table 2 shows detailed information regarding which Laird et al. (2011) networks were used, the labeling scheme used in the remainder of this paper, how many ROIs were assigned to each of these networks, and the color assigned to the nodes of each network in the result figures.

**Table 2:** **Summary of correspondence between Craddock Atlas ROIs and Laird Network Templates**.

**Original network ID (Laird etal., 2011)**	**New network ID**	**Number of ROIs**	**Description**	**Node color**	
ICN01	EI4	7	Emotion/Interoception network #4	Cyan	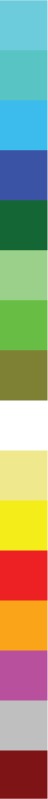
ICN02	EI3	10	Emotion/Interoception network #3	Aqua	
ICN03	EI2	6	Emotion/Interoception network #2	Light blue	
ICN04	EI1	8	Emotion/Interoception network #1	Dark blue	
ICN06	MV1	8	Motor/Visuospatial network #1	Dark green	
ICN07	MV2	8	Motor/Visuospatial network #2	Light green	
ICN08	MV3	5	Motor/Visuospatial network #3	Green	
ICN09	MV4	4	Motor/Visuospatial network #4	Olive green	
ICN10	VS1	9	Visual network #1	White	
ICN11	VS2	5	Visual network #2	Dark yellow	
ICN12	VS3	11	Visual network #3	Yellow	
ICN13	DMN	12	Default mode network	Red	
ICN15	FPR	13	Right fronto-parietal network	Orange	
ICN16	AUD	11	Auditory network	Pink	
ICN17	SPP	7	Speech production network	Gray	
ICN18	FPL	6	Left fronto-parietal network	Brown	

### ROI representative time series extraction

For each ROI, the principal singular vector (computed with AFNI program *3dmaskSVD*) across all voxels in the ROI was used as the representative time series. This resulted in 130 time series of interest with 3590 time points in each subject. The average and standard deviation of the Pearson's correlation between each ROI's representative time series and all voxels in the ROI, across all subjects and all ROIs, was 0.61 ± 0.08.

### Connectivity matrix based on whole time series: stationary analysis

For each subject, we computed an overall correlation matrix (130 × 130) under the assumption of temporal stationarity, using all available 3590 time points. In these matrices, connectivity between two given ROIs is measured in terms of their Pearson's correlation (*r*). These matrices are symmetric, with *r* = 1 along the diagonal. All information is therefore contained in the 8385 values that form the upper triangular region. In the remainder of this manuscript we use the term “connectivity snapshot” to refer to a vector that contains only these uniquely informative values.

Binarized (connected/not-connected) versions of these connectivity matrices were also obtained using the following criteria: a cell in the matrix is given a value of 1 (connected) only if the corresponding correlation value for that cell is statistically significant at *p* < 0.05 corrected for multiple comparisons according to the Bonferroni criteria, taking into account the number of unique connections in the matrix (i.e., *p* < 0.05/8385). Otherwise, the cell is given a zero (not-connected) in these binary matrices. Even though the correct DOFs (Table 1) were used when computing the significance of the correlations prior to the multiple comparison correction, the significance level is approximate due to the unknown relationship between signal and noise in rsfMRI.

### Selection of connections of interest for sliding window analysis

For our exploratory analysis of rsfMRI dynamics, we studied connections that showed significant correlation values in the stationary analysis for at least seven participants (half of the sample plus one). This selection step reduced the number of pairwise connections under study from the original 8385 to 5232 connections (see Figure 3).

### Whole-brain, within-subject connectivity matrix similarity vs. window duration

In order to evaluate how the within-subject similarity of whole-brain connectivity patterns changes as a function of window length, we segmented our 60 min of data (minus the first 10 discarded seconds) into temporally non-overlapping windows with durations ranging from 30 s to 19.5 min in steps of 30 s. The number of available non-overlapping windows decreases with increasing window duration. A maximum duration of 19.5 min was chosen so that at least three different windows were available for the analysis in each individual.

For each subject and window duration, we first computed connectivity matrices for each non-overlapping window. We then computed the average correlation between all available matrices for a given duration and subject. This average number permits us to describe within-subject similarity between connectivity matrices for a given duration. We finally computed an average value across all subjects, for each window duration, to obtain an aggregate measure of within-subject similarity for our population of subjects (Figure 4).

### Connection stability analysis

For each subject, we computed sliding window correlations with a window length of 60 s and a window step of 60 s (to avoid overlap). There are two reasons for choosing this 60 s window duration: (1) to have a sufficiently large number of data points per window to compute meaningful correlation values; and (2) because recent studies have shown that functional connectivity is related to both cognition (Shirer et al., 2012) and electrocortical measures (Tagliazucchi et al., 2012) at similar temporal scales. Nevertheless, to evaluate the extensibility of these results to other window durations, we also performed the same analysis using non-overlapping windows of 120 and 180 s durations.

**Figure 2:**
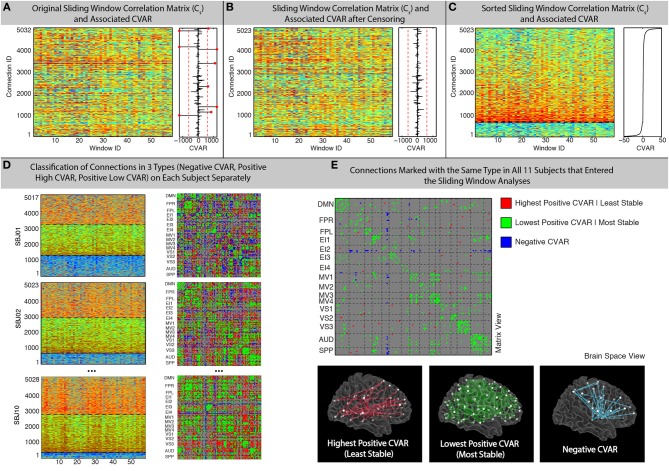
**Sliding-Window methods. (A)** Example running window connectivity matrix for one representative subject on the left, and its associated vector of CVAR values on the right. The thresholds used to discard connections on the basis of excessive CVAR are depicted as red dashed lines. Eight connections that were discarded for this particular subject are marked as red dots. **(B)** Sliding window connectivity matrix and CVAR vector after removal of outlier connections. Now there are 5023 connections, instead of 5032, for this representative subject. **(C)** Sliding window connectivity matrix and CVAR vector after sorting connections according to their CVAR. Connections with negative CVAR are at the bottom of the graph, while connections with positive CVAR are on the top. The further a connection is from the horizontal axis where CVAR is the closest to zero (black dashed line), the more variable the strength of that connection across time. **(D)** Classification of connections in three possible groups for three other representative subjects, shown both as sorted sliding window connectivity matrices (left) and in a single matrix form (right) where the color of the cell for a connection denotes its group assignment according to our criteria. The three groups are: connections with negative CVAR (blue); lowest positive CVAR/most stable connections (green); largest positive CVAR/least stable connections (red). **(E)** Aggregated results across subjects. We do this by only selecting connections classified the same way across all 11 participants that were included in the sliding window analysis. Connections of the three types are shown both in matrix view (top) and in brain space (bottom).

A 20% tapering of the time series was performed prior to computation of the correlation. For 60 s windows, the sliding window analysis produced for each participant (s) a matrix C_s_ (connection, window) with 5032 connections X 59 windows (not 60 due to the 10 s discarded at the beginning of the scan) that contains information about the evolution of connectivity strength over time for all connections under scrutiny (Figure 2A).

#### Most stable/variable connections

Subsequently, for each row of this matrix, we computed the coefficient of variation (CVAR) as follows:

(1) $$\text{CVAR }(\text{i},\text{​s})=\text{stdev }({\text{C}}_{\text{s}}(\text{i},\text{​}:\text{​}))/\text{mean }({\text{C}}_{\text{s}}(\text{i},\text{​}:))$$

where s is a given subject and i is a given connection (Figure 2A). In order to compute this summary metric we transformed correlation values into Fisher's *Z*-scores, computed the summary statistics, and then transformed these back from Fisher's *Z*-scores into correlation values.

In addition, the median and standard deviation of CVAR values across all subjects and connections was computed, and connections whose CVAR was outside one standard deviation of this median were removed from further analyses (Figure 2B). This threshold condition eliminated 9 ± 5 (mean ± standard deviation) connections per subject. After removal of outlier connections, the C_s_ matrices were sorted according to their CVAR values (Figure 2C). We then classified all remaining connections into one of three groups (Figure 2D). First, we divided the pool of connections into those with positive or negative CVAR. Then, within the pool of connections with positive CVAR, we further subdivided these into two subgroups: 50% of the positive CVAR connections with the highest CVAR values went into one subgroup (most variable), and the remaining half went into the other subgroup (most stable). In summary, this process forces every non-outlier connection to be part of one these three groups:

- *Negative Connections (blue)*: connections with negative CVAR, which is the result of a negative average Pearson's correlation across time.
- *Most Stable Positive Connections (green)*: connections in the lowest half of positive CVAR values.
- *Most Variable Positive Connections (red)*: connections in the highest half of positive CVAR values.

To aggregate results across subjects while giving maximum attention to connections with a similar pattern of correlation across participants, we generated a new group-level classification matrix in which a given connection was marked as being of one of the three types mentioned above, if and only if, that connection was classified in the same manner in all participants (Figure 2E—Top). In addition, to examine the effect of this threshold, matrices were also generated showing the number of subjects in which connections were classified in each group (Figure 7). To evaluate the presence of patterns of interest in the spatial distribution of these three types of connections, we used AFNI program *SUMA* (Saad and Reynolds, 2012) to visualize each of these three groups in a 3D brain space (Figure 2E—Bottom).

#### Permutation analysis for group-level connection identification

In order to determine the probability that results of the connection grouping procedure described above would occur due to chance, we conducted a permutation test in which the labels of all connections in each subject were randomly shuffled. Using the same group sizes for each subject from the real data, the connections for each group were then selected within that subject. The number of connections classified in the same group across all subjects was then counted. This procedure was repeated 5000 times to obtain a distribution of the number of connections that would be classified in the same group in all subjects based only on chance.

## Results

### Stationary analyses results

**Figure 3:**
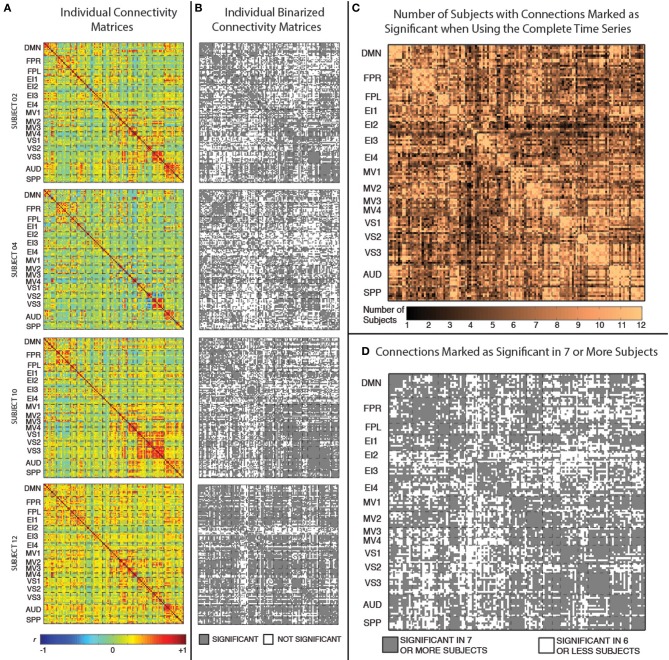
**(A)** Weighted connectivity matrices in terms of Pearson's correlation (*r*) for four representative subjects when the complete time series (3950 data points) enter the analysis. **(B)** Binary connectivity matrices for the same four representative subjects after statistical thresholding. **(C)** Matrix showing the number of subjects for which a given connection was marked as statistically significant. **(D)** Matrix showing connections that were marked as statistically significant in at least seven subjects.

Figure 3A shows the static connectivity matrices for four representative subjects computed using the complete time series (3590 time points). Although there is some degree of similarity in the overall structure of the matrices across subjects (e.g., within-network connections are stronger than between-network connections in all subjects; connectivity between MV2 and VS3 is also stronger in many subjects), there are clear differences in terms of the strength of many individual connections. From a quantitative point of view, the average correlation between the different subjects' connectivity snapshots (upper top triangle of the matrix excluding the diagonal) is *r* = 0.53 ± 0.07.

Figure 3B shows binarized (connected/unconnected) versions of the connectivity matrices presented in Figure 3A. The average and standard deviation number of statistically significant connections for the current sample was 5198 ± 747 (out of 8385 possible connections). Figure 3C shows another matrix view of the data where the value in each cell is the number of subjects for which that particular connection is statistically significant under the criteria described above. Finally, Figure 3D shows a binarized version of this aggregate view (Figure 3C), by marking with gray color only the connections that were classified as statistically significant in at least seven (more than half of the study population) subjects. There are a total of 5032 connections that pass this group-level threshold. All remaining results, with the exception of the whole-brain within-subject similarity vs. scan duration analysis (section Similarity of Whole-Brain Connectivity as a Function of Window Duration), were conducted using only this subset of 5032 connections.

### Similarity of whole-brain connectivity as a function of window duration

**Figure 4:**
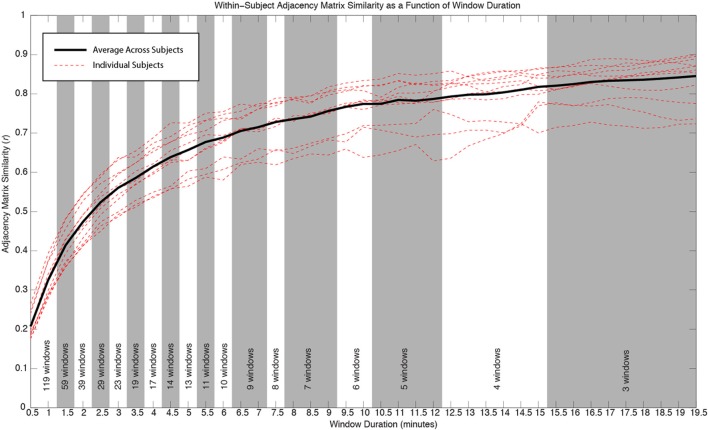
**Similarity of whole-brain connectivity vs. window duration**. Data for individual subjects are shown as red dashed lines. Average across all subjects is shown in black. The similarity of connectivity matrices clearly decreases as a function of window duration. The decreasing rate is particularly accentuated for durations below 10 min. As window duration decreases, a larger number of windows enter the analysis for a given duration. The number of windows contributing to each duration in each subject is provided at the bottom of the graph. All window durations within the same shaded region (white or gray) have the same number of windows contributing to the computation of similarity.

Figure 4 shows how within-subject similarity of connectivity patterns across the whole brain decreases as a function of window duration. For durations larger than 10 min, the rate of decrease is relatively slow. It is for durations shorter than approximately 6 min that within-subject similarity decreases at a faster rate. This behavior was consistent across subjects.

### Histograms of sliding-window correlations

**Figure 5:**
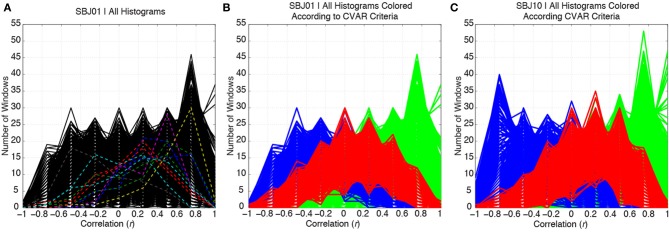
**(A)** Distributions of correlation values across time for all the connections in a representative subject are depicted in black. To highlight the mostly uni-modal shape of individual histograms, 11 randomly selected histograms are highlighted using dashed colored lines. **(B)** Same histograms as in **(A)**, but this time each histogram is colored according to the membership of each connection to one of three groups: blue = negative CVAR connection; red = most variable positive CVAR connection; green = most stable positive CVAR connection. Grouping of connections show a compact profile with all connections from the same group clustering together. **(C)** Same as **(B)** for a second representative subject.

Figure 5A shows histograms of correlation values across time (bin width = 0.25) for all connections in one representative subject (SBJ01) as black traces. Visual inspection reveals no clear boundaries between different connection types, but a continuum of behavior in which connections span a wide range of mean and standard deviation values. Peaks can be observed at all centers of histogram bins. This is not the result of individual histograms having many peaks (temporal evolution of connectivity strength following multimodal distributions), but due to the overlap of approximately 5000 histograms with a wide range of means and standard deviations. To show how individual histograms do not present such sharp profiles, but are mostly uni-modal in shape, a subset of 11 randomly selected histograms are highlighted with dashed colored lines in Figure 5A. Figure 5B shows the same histograms as Figure 5A, but this time histograms have been colored according to their membership to one of the three groups defined in terms of CVAR (blue = negative CVAR; red = most variable positive CVAR; green = most stable positive CVAR). Despite the lack of clear boundaries between histograms, the classification criteria based on the CVAR were able to generate three compact groups of connections in all subjects (Figure 5C shows a second representative subject). An additional observation is that most stable positive connections, as defined with the CVAR criteria, are connections with high mean connection strength across time (green histograms peak primarily at the right of the graphs).

### Most variable positive connections

**Figure 6:**
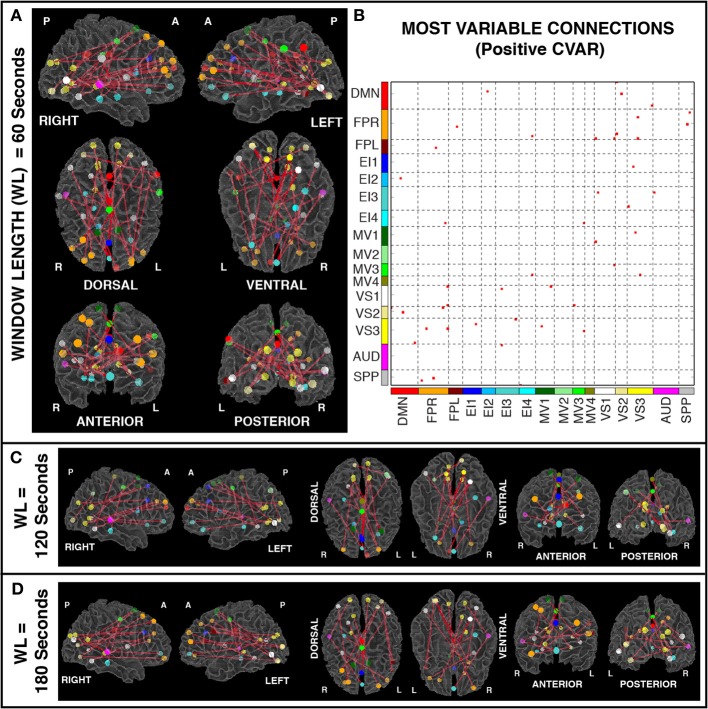
**(A)** Most variable positive connections for window length = 60 s. Connections classified as most variable in all 11 participants are shown over 3D renderings of a brain surface. **(B)** The same information shown as a 2D matrix. Colors corresponding to networks on the axes of the matrix are used to color nodes of that network in brain space. **(C)** Most variable connections for window length = 120 s. **(D)** Most variable connections with window length = 180 s.

Figures 6A,B show the 23 connections classified as most variable in all participants for a window duration of 60 s. Table 3 summarizes the distribution of such connections across different networks. All 23 connections correspond to connections between ROIs from different networks (Table 3). Primarily, most variable connections correspond to non-symmetric, inter-hemispheric connections between occipital (visual networks) and frontal regions (fronto-parietal networks). A similar general pattern was observed for window durations of 2 (Figure 6C) and 3 (Figure 6D) min. The total number of connections in this pool was 13 for 2 min windows, and 14 for 3 min windows.

**Table 3:** **Absolute (#) and relative (%) number of connections with positive high CVAR (most variable) for each network**.

**Most variable**		**Network ID**															
		**DMN**	**FPR**	**FPL**	**EI1**	**EI2**	**EI3**	**EI4**	**MV1**	**MV2**	**MV3**	**MV4**	**VS1**	**VS2**	**VS3**	**AUD**	**SPP**
Within	#	0															
	%																
Across	#	4	9	1	1	1	3	3	2	0	3	0	5	3	7	1	3
	%	0.47	1.09	0.21	0.16	0.37	0.49	0.68	0.31	0.00	0.79	0.00	0.77	1.06	0.90	0.12	0.56

*Connection counts are divided in two groups: connections between two ROIs that are part of the same network (within) and connections between ROIs that are part of different networks (across)*.

**Figure 7:**
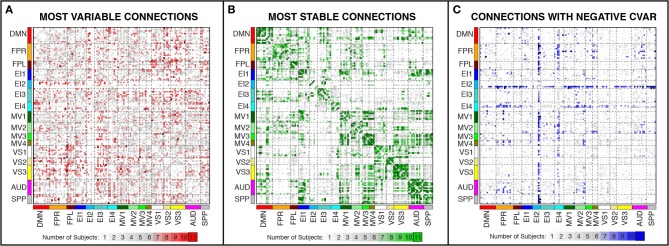
**Number of subjects for which a given connection was classified as most variable (A), most stable (B), and with negative CVAR (C)**. Connections that were consistently classified in the same group for all 11 subjects are marked with a black outline. These are the same connections shown in Figure 6 (most variable), Figure 8 (most stable), and Figure 11 (negative CVAR). Connections that were classified in the same group for seven or more subjects appear in different shades of red (most variable), green (most stable), or blue (negative CVAR) in the corresponding panel.

In addition, Figure 7A shows a non-thresholded version of Figure 6B, where the color of each connection represents the number of subjects for which that connection was classified as most variable. Connections marked as most variable for seven or more subjects are colored with different shades of red. These connections still correspond primarily to inter-network connections. Moreover, they tend to correspond primarily to connections between occipital (visual networks) and fronto-parietal networks, as well as connections between nodes of EI3 and all other networks.

### Most stable positive connections

**Figure 8:**
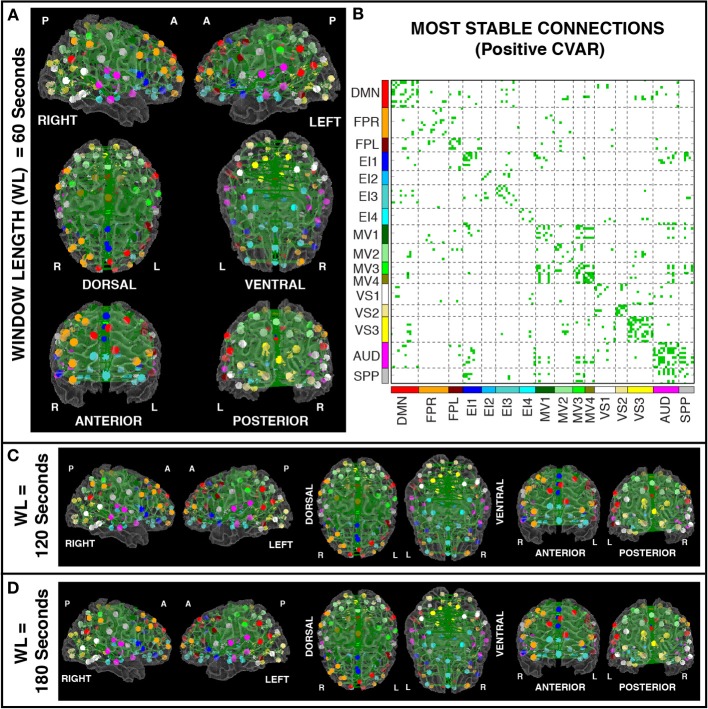
**(A)** Most stable positive connections for window length = 60 s. Connections classified as most stable in all 11 participants are shown over 3D renderings of a brain surface. **(B)** The same information shown as a 2D matrix. Colors corresponding to networks on the axes of the matrix are used to color nodes of that network in brain space. **(C)** Most stable connections for window length = 120 s. **(D)** Most stable connections with window length = 180 s.

**Figure 9:**
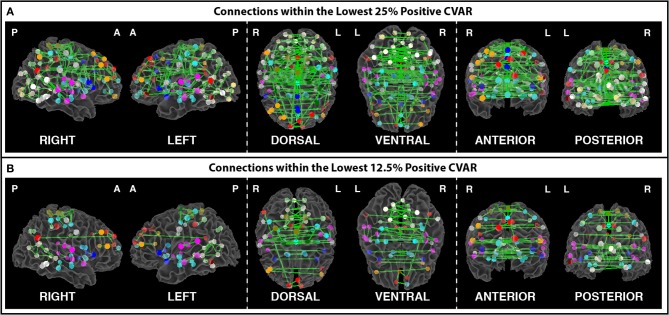
**(A)** Most stable positive connections when only connections within the lowest 25% of CVAR values are selected in each subject. **(B)** Most stable positive connections when only connections within the lowest 12.5% of CVAR values are selected in each subject. As the selection criterion becomes more stringent, a smaller number of connections make it to the group level maps presented here. When fewer connections are present, the symmetric inter-hemispheric pattern becomes clearer.

Figures 8A,B show the 364 connections classified as most stable in all participants for a window duration of 60 s. Table 4 summarizes the distribution of these connections within and across different networks. Roughly 40% of the connections, 148, correspond to within-network connections and the remaining 216 to across-network connections. A large percentage of stable positive connections are symmetric, inter-hemispheric connections. This pattern becomes more apparent if we restrict our analysis only to connections in the bottom 25% and 12.5% of positive CVAR values (Figure 9). When window duration was increased to 2 (Figure 8C) and 3 (Figure 8D) min, a similar spatial pattern arises. The total number of positive stable connections was 344 for 2 min windows, and 334 for 3 min windows.

**Table 4:** **Absolute (#) and relative (%) number of connections with positive low CVAR (most stable) for each network**.

**Most stable**		**Network ID**															
		**DMN**	**FPR**	**FPL**	**EI1**	**EI2**	**EI3**	**EI4**	**MV1**	**MV2**	**MV3**	**MV4**	**VS1**	**VS2**	**VS3**	**AUD**	**SPP**
Within	#	23	9	6	9	2	8	3	7	5	5	6	4	7	26	25	3
	%	44.23	12.50	40.00	37.50	28.57	22.86	15.00	26.92	17.86	50.00	100.00	11.76	70.00	47.27	50.00	14.29
Across	#	44	15	18	34	3	20	14	38	22	40	25	25	4	28	60	42
	%	5.15	1.82	3.79	5.48	1.11	3.24	3.19	5.94	3.53	10.58	8.09	3.86	1.41	3.60	7.13	7.87

*Connection counts are divided in two groups: connections between two ROIs in the network of interest (within) and connections between one ROI in the network of interest and one ROI not in the network of interest (across)*.

In addition, Figure 7B shows a non-thresholded version of Figure 8B, where connections classified as most stable for seven or more subjects appear with different shades of green. Most stable connections under these less stringent conditions correspond primarily to within-network connections, although several clusters of most stable connections can be observed between the AUD and SPP networks, between the four MV networks, and between MV3-4 and visual and auditory regions.

**Figure 10:**
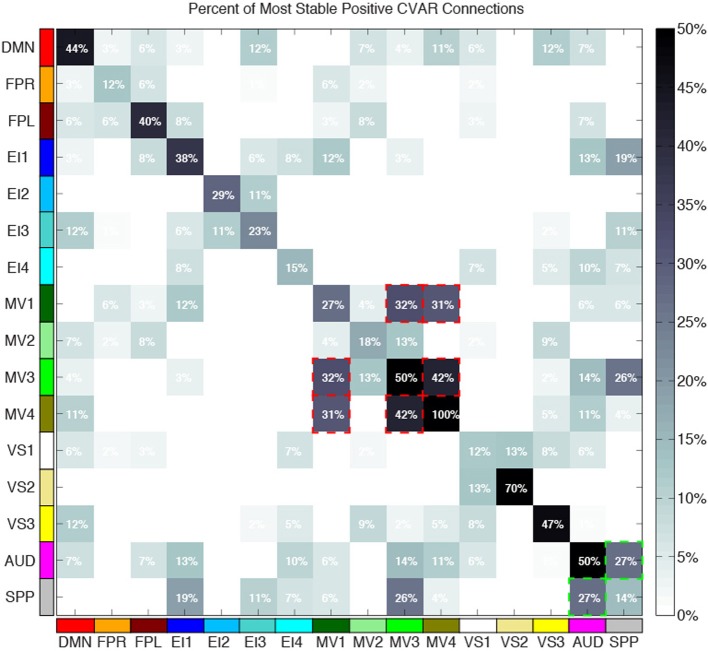
**Percentage of within-network (diagonal) and across-network (non-diagonal) most stable positive connections**. Two groups of networks with high percentages of across-network connections being temporally stable are highlighted with red (MV1, MV2, and MV3) and green (AUD, SPP) dashed lines.

Figure 10 shows a summary view of the matrix in Figure 8B. For each square, we show the percentage of connections that fall within the most stable positive pool. Therefore, squares in the diagonal show the percentage of within-network connections that were classified as most stable. For example, MV3 and VS2 are the two most cohesive networks, with 100 and 70% of all possible within-network connections being consistently stable across time. Squares outside the diagonal show the percentage of all possible connections between two given networks that fall within the pool of most stable connections. We can see how MV1, MV3, and MV4 (red dashed outlines) have a substantial number of stable communication pathways among each other. The same is true for the SPP and the AUD networks (green dashed outlines). All percentages in this figure have been corrected to take into account only the 5032 connections that passed our stationary significant criteria.

### Negative connections

**Figure 11:**
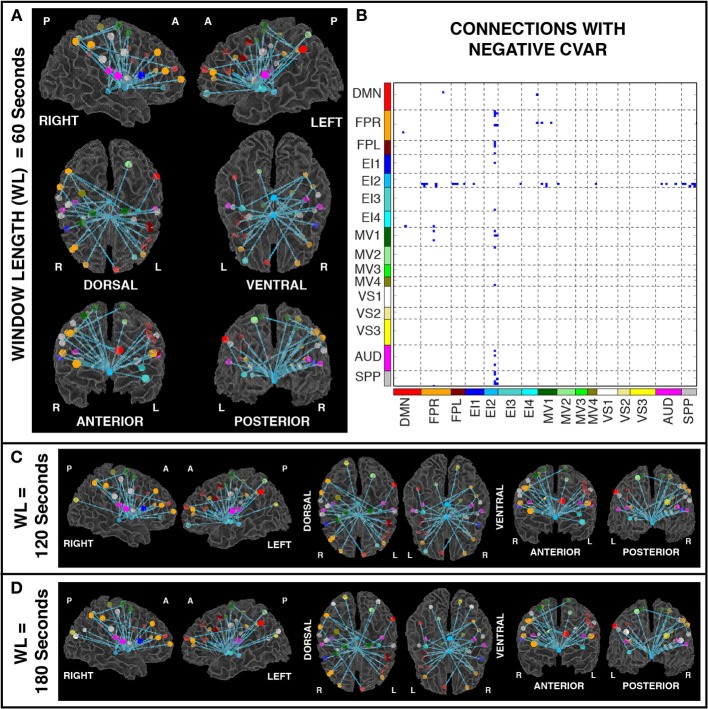
**(A)** Negative CVAR connections for window length = 60 s. Connections with negative CVAR in all 11 participants are shown over 3D renderings of a brain surface. **(B)** The same information shown as a 2D matrix. Colors corresponding to networks on the axes of the matrix are used to color nodes of that network in brain space. **(C)** Negative CVAR connections for window length = 120 s. **(D)** Negative CVAR connections with window length = 180 s.

Figures 11A,B show the 32 connections with negative CVAR in all participants for a window duration of 60 s. Table 5 summarizes the distribution of such connections across different networks. All negative connections correspond to across-network connections. In particular, 26 connections involve two regions from the Emotion/Interoception network #2 (EI2). This pattern of negative CVAR connections primarily involving regions from the EI2 network is also very apparent in Figure 7C, where connections marked as negative CVAR connections in seven or more subjects appear marked in different shades of blue. When window duration was increased to 2 (Figure 11C) and 3 (Figure 11D) min a similar connectivity map was also produced. The total number of negative connections was 32 for 2 min windows, and 30 for 3 min windows.

**Table 5:** **Absolute (#) and relative (%) number of connections with negative CVAR for each network**.

**Negative**		**Network ID**															
		**DMN**	**FPR**	**FPL**	**EI1**	**EI2**	**EI3**	**EI4**	**MV1**	**MV2**	**MV3**	**MV4**	**VS1**	**VS2**	**VS3**	**AUD**	**SPP**
Within	#	0															
	%																
Across	#	2	11	4	1	26	1	2	5	1	0	1	0	0	0	3	7
	%	0.23	1.34	0.84	0.16	9.63	0.16	0.46	0.78	0.16	0.00	0.32	0.00	0.00	0.00	0.36	1.31

*Connection counts are divided in two groups: connections between two ROIs in the network of interest (within) and connections between one ROI in the network of interest and one ROI not in the network of interest (across)*.

## Discussion

Using 60 min resting scans with a temporal resolution of 1 s and a sliding window analysis approach, we divided functional connections in our data into three groups based on similarity of patterns of temporal variability across our study population. Sorting and grouping of connections was done according to the coefficient of variance (CVAR) of connectivity strength across time. The CVAR is a common measure of spread for Gaussian-like distributions that accounts for differences in the mean and has a simple interpretation (i.e., the larger the CVAR, the bigger the spread of the distribution of values around the mean). Connectivity strength histograms (Figure 5) showed distributions follow mostly uni-modal, bell-like shapes with different levels of spread, suggesting that the use of CVAR is a valid first approximation to estimate variability for the temporal evolution of connection strength. To aggregate results at the group level, we decided to focus our attention only on connections classified in the same manner across all participants. A permutation analysis (5000 repetitions) revealed that the number of connections randomly found in any of the three groups, when following the above-mentioned criteria to combine results across subjects, is less than four connections. Finally, to evaluate the role that regional differences in signal-to-noise ratios may have played in our study, we also computed average temporal signal-to-noise ratio (TSNR) across subjects for all ROIs entering the analysis. We found no clear relationship between ROI TSNR values and participation in connections of a given type (most variable, most stable, or negative CVAR). These results suggest that the simple criteria used in this study provide reasonable descriptions of the patterns of temporal variability in resting state connectivity, and that these results are reproducible across subjects and capture true structure present in the data (i.e., not found by purely by chance).

The connections that reliably fall in each category have very distinct spatial patterns when plotted in brain space. In particular, most temporally stable connections (low positive CVAR) correspond mainly to symmetric, inter-hemispheric connections both within- and across-networks; most temporally variable connections (high positive CVAR) correspond mainly to non-symmetric, inter-hemispheric, across-network connections between occipital and frontal regions; and connections with negative CVAR correspond mainly to connections between two medial ventral subcortical regions and bilateral fronto-parietal regions. These general patterns were observed for non-overlapping window durations ranging from 1 to 3 min. We discuss the findings related to each of these categories in detail below.

### Most stable positive connections

Most stable positive connections is the largest of the three connection pools, with approximately one order of magnitude more connections than the other two groups (364 most stable connections vs. 23 and 32 in the other two groups). Moreover, most stable connections are not only more consistent across subjects and fluctuate less, but fluctuate around higher correlation values than least stable connections (green histograms cluster on the right hand side, which corresponds to stronger positive correlation values; see Figures 5B,C). These two observations suggest that while being classified as most variable or negative may depend to a larger extent on subject-dependent factors (e.g., on-going cognition, awareness levels, etc.), most stable connections are so because of an underlying source largely independent of these factors. One such source could be anatomical connectivity. Several studies have shown a good correspondence between BOLD resting state connectivity patterns and underlying direct anatomical connections as measured in Diffusion Tensor Imaging (DTI) (Greicius et al., 2009; Van Den Heuvel et al., 2009) and in primate electrophysiology and tracer studies (Margulies et al., 2009; Wang et al., 2013b). Additionally, computational modeling studies have shown that structural connections provide robust predictions of functional connectivity, although the reverse is not always true (Honey et al., 2009; Deco et al., 2011). Relating to the current study, Honey et al. (2009) observed that ROI pairs with direct anatomical connectivity—as measured by diffusion spectrum imaging tractography—had more stable functional connectivity both within and across rsfMRI sessions. In agreement with their findings, many of the most stable connections identified here are symmetric, inter-hemispheric connections between left/right homologous regions that are known to have direct connections via the corpus callosum. However, it should also be noted that stable functional connectivity patterns can also be supported by indirect anatomical connections as well (Tyszka et al., 2011; O'Reilly et al., 2013).

Approximately 40% of the most stable connections correspond to those between two nodes of the same network (within-network connections). Still, that accounts for only 32% of all within-network connections, which confirms prior observations suggesting that resting-state networks are not as temporally stable in their configuration as originally assumed (Chang and Glover, 2010; Handwerker et al., 2012; Smith et al., 2012; Tagliazucchi et al., 2012; Hutchison et al., 2013b). Our data also shows that levels of temporal cohesion vary substantially across networks. The four most temporally cohesive networks were MV4 (100% of its 6 within-network connections fall in the most stable group), VS2 (70%), MV3 (50%), and AUD (50%) (Figure 10 and Table 4). The MV4 network, which primarily covers bilateral dorsal parietal cortex (BA5), has been shown to have a preference for motor execution and learning (Laird et al., 2011). The MV3 network, which sits laterally to MV4 and covers mainly primary and supplementary motor cortex for upper extremities was found to be strongly associated with tasks involving hand movement (Laird et al., 2011). Additionally, networks VS2 (which covers posterior and inferior portions of occipital cortex) and AUD (which covers the transverse temporal gyri) correspond to primary visual and auditory cortices. Taken together, our results suggest that primary sensory-motor networks are among the most temporally stable with respect to their internal connectivity patterns. On the other end of the spectrum, VS1 (11.76%), FPR (12.50%), SPP (14.20%), and EI4 (15%) were the networks with the lowest percentage of within-network connections that were consistently stable across all subjects. These networks span a wide range of regions involved in complex higher-order functions such as visual identification of complex visual stimuli (VS1), attention control and reasoning (FPR), speech production (SPP), and emotion discrimination (EI4). It may be that performance of these more complex tasks relies on a broader and more dynamic set of connectivity configurations, and that these tasks and their configurations occur less often during rest. In agreement with these findings, Mueller et al. (2013) found that inter-subject variability in stationary patterns of global functional connectivity was lowest in unimodal cortical areas similar to the sensory-motor systems found to be most stable here.

Regarding most stable between-network connections, we found two sets of networks to be the most stably interconnected. The first group consists of networks MV1, MV3, and MV4 (red outlines in Figure 10). The second group consists of SPP and AUD (green outlines in Figure 10). These groups of networks were found to be tightly connected in terms of their functional role when matched against thousands of activity patterns from task-based studies included in the BrainMap database (Fox et al., 2005). MV1, MV3, and MV4 were found to consistently participate in a variety of experiments related to motor and visuo-spatial integration and coordination (Laird et al., 2011). Moreover, MV3 and MV4 (the two networks with the largest percentage of inter-network stable connections) failed to split into two separate entities in a prior similar study that used a smaller subsample of the BrainMap database (Smith et al., 2009). In the case of the SPP and AUD networks, their functional relationship was not as strong, but both networks heavily contribute to language-related tasks. These reported agreements between network groupings based on functionality (as measured by paradigm and behavioral domain) and levels of stable inter-connectivity suggest that networks that share a common functional space (e.g., motor-visual integration, language) also share stable communication pathways, despite appearing as separate entities in resting state analyses that do not focus on the dynamic aspects of connectivity. Nonetheless, it is worth noticing that the other two multi-network functional spaces defined by Laird et al. (2011), namely emotion/interoception and visual, did not show such a clear pattern of stable interconnectivity between networks.

### Most variable positive connections

Most variable positive connections correspond primarily to inter-network, inter-hemispheric connections involving nodes from the fronto-parietal networks (FPR: 9 connections; FPL: 1 connection) and the visual networks (VS3: 7 connections; VS2: 3 connections; VS1: 5 connections). It has been previously shown that the fronto-parietal network is composed of flexible hub regions that can reconfigure their functional connectivity rapidly in order to adapt and participate in a great variety of externally driven tasks (Cole et al., 2013). Our results suggest that such flexibility can also be observed during undirected cognition while resting, and not solely in situations requiring highly adaptive task control. Moreover, a recent study showed that subjects engage and transition between many different mental activities while resting in the scanner (Delamillieure et al., 2010). The three most common mental activities reported by this pool of 180 subjects were visual imagery, inner speech, and somatosensory awareness. All but one across-network connections involving the fronto-parietal network also involve nodes from the visual and SPP networks, which are directly related to these mental activities commonly reported by subjects after rest scans. Lastly, additional connections belonging to this category outside the fronto-parietal network correspond primarily to connections between occipital regions and nodes from the DMN, motor/visuospatial networks, and the emotion/interoception networks (as described by Laird et al.). Some of these areas, in particular DMN and heteromodal occipital regions, overlap with areas described as part of the “Zone of Instability” (regions with more temporally variable connections between them) by Allen et al. (2014).

Although high temporal variability makes these connections a difficult target for study, the fact that such high volatility was consistent across all subjects in our pool suggests that these connections may constitute good targets for some technical and clinical applications. First, the pool of 23 connections identified as most variable across all subjects may constitute a good set of “worse-case scenario” targets for reproducibility studies and/or optimization of parameters such as scan duration. They could help obtain conservative bound values for such parameters. Moreover, the ability of certain regions to flexibly reconfigure their connectivity patterns has been shown to be directly related to the capacity to learn new motor skills (Bassett et al., 2011). Finally, Mueller et al. (2013) recently showed that areas with the largest levels of inter-subject variability in stationary global connectivity patterns correspond primarily to heteromodal association cortex in lateral pre-frontal cortex, the temporal-parietal junction, fronto-parietal control regions, and attention network areas (as defined by Yeo et al., 2011). They also reported a large degree of overlap between these regions of high functional connectivity variability and a brain map obtained from a meta-analysis of areas that predict individual differences in several cognitive and behavioral domains (e.g., personality traits, intelligence, memory performance, etc.) Many of the connections classified as most variable in our study are between ROIs located in the areas and networks of high variability reported by Mueller and colleagues. This suggests that short-term temporal variability in connectivity patterns (as observed here) may be partially responsible for the inter-subject differences in functional connectivity observed at longer temporal scales, which may in turn be related to individual differences in cognition and behavior. Given the consistently high temporal instability of these connections across all our healthy subjects, it would be interesting to study if temporal variability is somehow impaired or increased in populations with some level of cognitive decline, and in that manner evaluate the potential diagnostic power of the dynamic behavior of rsfMRI connectivity.

### Negative connections

**Figure 12:**
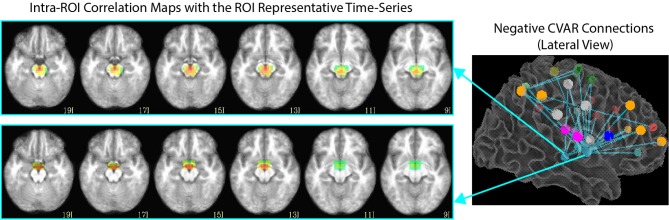
**Intra-ROI correlation maps to the ROI representative time-series for 2 ROIs of interest**. Voxels with the highest correlation to the representative time series (red color) are those in and around the third ventricle.

Of the 32 connections with negative CVAR in all participants, 26 correspond to connections involving two medial ROIs that are part of the EI2 network. The first ROI (with 21 negative connections) spans a large range of small anatomical structures, including the mammillary bodies, the hypothalamus, medial portions of the caudate, the fornix, and the third ventricle. The second ROI (with 5 negative connections) is located just posterior to the first and covers large portions of the bilateral thalamus. Correlation maps between each ROI's representative time series and all ROI voxels (Figure 12) show how the highest contributing voxels to the representative time series fall primarily within or around the third ventricle. This is particularly true for the ROI with 21 negative CVAR connections. This pattern suggests that negative correlations between these ROIs and other brain regions are not the result of anti-correlation between GM structures within the ROIs and other brain regions, but a result of the regression of CSF signals during pre-processing (Saad et al., 2012). In this study, the CSF signals may have been contaminated by signals from other neighboring tissues due to the relatively large voxel size used in this study. In fact, when the removal of CSF signal is omitted from the analysis pipeline, only three connections with negative CVAR remain, thereby supporting the potential artifactual origin of the average negative behavior observed for these connections. Conversely, the general patterns described for the other two connection types (most stable and most variable) remains consistent when CSF is not removed during the analysis.

It is also worth noting that while omitting the step concerning the removal of CSF signals led to the disappearance of the majority of connections with an average negative correlation (and therefore negative CVAR), we nevertheless observed many connections alternating between positive and negative connectivity for short periods, regardless of CSF signal removal. This is in agreement with prior observations of this phenomenon in studies on functional connectivity dynamics (Chang and Glover, 2010; Hutchison et al., 2013b).

### Stability of within-subject connectivity patterns vs. window duration

In addition to classifying connections in the three above-mentioned groups, we also evaluated how window length (used here as a proxy for scan duration) affects the within-subject similarity of whole-brain connectivity patterns. We found two general regimes. For durations below approximately 6 min, similarity of within-subject whole-brain connectivity matrices decreases quickly as window length decreases. Conversely, for durations above 10 min, the rate at which similarity increases with scan duration is much slower. This result suggests that if stability is a factor of interest (e.g., in longitudinal studies), using longer scans is desirable, particularly above approximately 10 min. Most previous studies of rsfMRI reproducibility have used shorter scans and focused on a handful of connections when evaluating the temporal stability of rsfMRI as a function of scan duration. Van Dijk et al. (2010) concluded that stable measures of connectivity can be obtained with scans as short as 5 min. This conclusion was based on how scan duration affected average within- and between-network correlations for only three networks (default mode, dorsal attention, and a reference network consisting of auditory, motor, and visual regions). Nevertheless, Birn et al. (2013) more recently concluded that increasing scan length from 5 to 13 min greatly improved reproducibility. In this case, the authors studied all potential connections between 17 different ROIs. Using a completely different approach, Anderson et al. (2011) found that obtaining functional connectivity “fingerprints” that uniquely identified each participant required a minimum of approximately 15 min of data. Despite differences in scanning and analytical procedures, our results are in better agreement with those of Anderson et al. (2011) and Birn et al. (2013), which are based on larger samples of connections. This suggests that a minimum of approximately 10 min is desirable for good reproducibility, and that reproducibility keeps increasing at a lower rate for yet longer scan durations. Collectively, these results also highlight how suggested scan duration will depend on the target networks under analysis.

### Limitations of the study

In this study we did not record any measure of vigilance (e.g., eye tracking system, concurrent EEG recordings). Given the duration of the scans and that subjects were instructed to keep their eyes closed, it is very likely that our subjects went through some periods of sleep or decreased vigilance during the 60 min scans, despite being instructed to stay awake. Changes in vigilance or sleep are known to affect connectivity patterns measured with fMRI (Horovitz et al., 2009; Tagliazucchi et al., 2012). To partially evaluate the effect of this potential confound, we performed the analysis again using the first and last halves of the time series separately, under the assumption that periods of drowsiness will become more frequent as scanning progresses. When the data was split in this manner, the spatial patterns of connectivity per connection category and the bulk differences in number of connections per category remain very similar to those reported for the whole-run analysis (see Supplementary Figure 1). This suggests that although the classification of specific connections may be affected by this factor, the overall patterns discussed above remain present. Nevertheless, a better-controlled experiment with information about when these changes in vigilance occur may help better elucidate the origin of the patterns observed here. Also, restricting the analysis to periods of equal vigilance levels may help increase the number of patterns found to be common across subjects.

Another important factor to consider is how ROI and network templates used during the analysis affect interpretation of the data. We used a functionally-based atlas for the purpose of aggregating voxels into functionally homogenous regions. Functionally-based atlases have been proven to outperform anatomically-based atlases at reproducing functional connectivity patterns present at the voxel level (Craddock et al., 2012) and when attempting to decode cognitive states based on measures of connectivity (Shirer et al., 2012). In particular, the 150 ROI atlas was selected because it provided a good compromise between ROI size (sufficient functional homogeneity), computational tractability, and interpretability of the results. Using more fine-grained ROIs may allow detection of additional patterns of interest, and additional studies should be conducted to evaluate the robustness of the results presented here against the use of different parcellation schemes (Yeo et al., 2011; Shirer et al., 2012).

In a similar manner, the Laird et al. (2011) ICN templates were chosen to aid with interpretation given their behavioral correlates. Our discussion regarding the temporal stability of within- and across-network communication pathways heavily relies on the assignment of ROIs to these networks. Differences in network definition, and subsequent distribution of ROIs across them, may affect the conclusions. As of today, the fMRI community still debates which is the most informative decomposition level, or levels, to study resting state connectivity, as the configuration of networks heavily depends on this parameter (Abou-Elseoud et al., 2010). Moreover, there is an avid debate regarding the actual configuration of the well-studied default mode network (Buckner et al., 2008; Liu and Duyn, 2013). Comparative analyses between measures of temporal stability, such as the ones presented here, and network definitions obtained at different decomposition levels may help determine the most appropriate levels of brain parcellation.

## Conclusions

We used a sliding window analysis to attempt a basic characterization of BOLD resting state connectivity dynamics. We found three well-differentiated sets of connections, whose temporal variability patterns were reproducible across all participants and have distinct spatial patterns. First, most stable connections were found to correspond primarily to symmetric, inter-hemispheric connections both within and across networks. We found that primary sensory-motor networks seem to be more temporally stable in their connectivity patterns than those more closely related to higher order cognitive processes. Second, most variable connections were found to correspond primarily to non-symmetric, inter-hemispheric, across-network connections between occipital and frontal regions. The number of connections consistently among the most variable group across all subjects was much lower than the number of connections among the most stable, suggesting subject-dependent, ongoing cognitive variables have a strong effect on the configuration of flexible connections in the brain. Finally, a small set of connections was found to have negative average connectivity across time, though a large percentage of these were identified as potential artifacts. All these general patterns were present for window lengths ranging from 1 to 3 min.

We also used the current dataset to evaluate how whole-brain, within-subject similarity of connectivity patterns varies as a function of window duration. This applies to studies where the focus is not on the dynamic behavior of connections, but on overall stable patterns that arise when full scans enter the analysis. Our results suggest that in order to maximize similarity of overall whole-brain connectivity, rest scans should last as long as possible, with clear stability benefits for 10 min rather than 5 min scans.

### Conflict of interest statement

The authors declare that the research was conducted in the absence of any commercial or financial relationships that could be construed as a potential conflict of interest.
