# Supplementary material for: The spatial structure of resting state connectivity stability on the scale of minutes
Source: Front Neurosci. 2014 Jun 11;8:138. doi: 10.3389/fnins.2014.00138 (PMC4052097; doi:10.3389/fnins.2014.00138)
Supplement: Supplementary file 1 [file Presentation1.PPTX]

## Slide 1
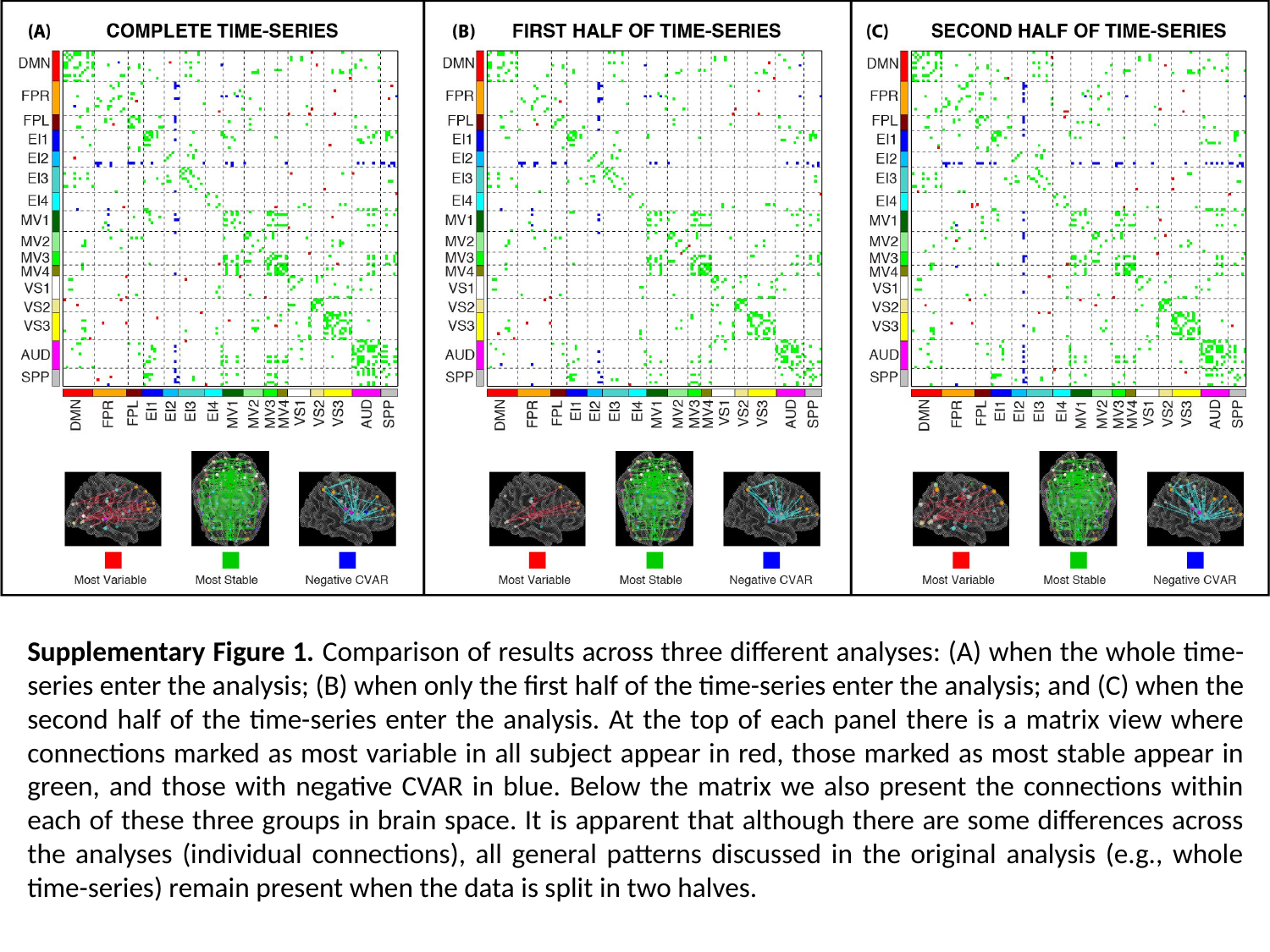

Supplementary Figure 1. Comparison of results across three different analyses: (A) when the whole time-series enter the analysis; (B) when only the first half of the time-series enter the analysis; and (C) when the second half of the time-series enter the analysis. At the top of each panel there is a matrix view where connections marked as most variable in all subject appear in red, those marked as most stable appear in green, and those with negative CVAR in blue. Below the matrix we also present the connections within each of these three groups in brain space. It is apparent that although there are some differences across the analyses (individual connections), all general patterns discussed in the original analysis (e.g., whole time-series) remain present when the data is split in two halves.
